# G-protein-coupled receptor 30-mediated antiapoptotic effect of estrogen on spinal motor neurons following injury and its underlying mechanisms

**DOI:** 10.3892/mmr.2015.3601

**Published:** 2015-04-08

**Authors:** JINGYU CHEN, RONG HU, HONGFEI GE, WANGSHENG DUANMU, YUHONG LI, XINGSENG XUE, SHENGLI HU, HUA FENG

**Affiliations:** Department of Neurosurgery, Southwest Hospital, Third Military Medical University, Chongqing 400038, P.R. China

**Keywords:** estrogen, motor neuron, G-protein-coupled receptor 30, apoptosis, phosphatidylinositol 3-kinase/protein kinase B

## Abstract

Spinal cord injury (SCI) may result in severe dysfunction of motor neurons. G-protein-coupled receptor 30 (GPR30) expression in the motor neurons of the ventral horn of the spinal cord mediates neuroprotection through estrogen signaling. The present study explored the antiapoptotic effect of estrogen, mediated by GPR30 following SCI, and the mechanisms underlying this effect. Spinal motor neurons from rats were cultured *in vitro* in order to establish cell models of oxygen and glucose deprivation (OGD). The effects of estrogen, the estrogen agonist, G1, and the estrogen inhibitor, G15, on motor neurons were observed using MTT assays. The effects of E2, G1 and G15 on spinal motor neuron apoptosis following OGD, were detected using flow cytometry. The role of the phosphatidylinositol 3-kinase/protein kinase B (PI3K/Akt) inhibitor, LY294002, was also determined using flow cytometry. Rat SCI models were established. E2, G1 and E2+LY294002 were administered *in vivo*. Motor function was scored at 3, 7, 14, 21 and 28 d following injury, using Basso-Beattie-Bresnahan (BBB) standards. Cell activity in the estrogen and G1 groups was higher than that in the solvent group, whereas cell activity in the E2+G15 group was lower than that in the E2 group (P<0.05). Following OGD, the proportion of apoptotic cells significantly increased (P<0.05). The proportion in the estrogen group was significantly lower than that in the solvent group, whereas the proportion of apoptotic cells in the E2+G15 and E2+LY294002 groups was higher than that in the E2 group (P<0.05). Treatment with E2 and G1 led to upregulation of P-Akt expression in normal cells and post-OGD cells. The BBB scores of rats in the E2 and G1 groups were higher than those in the placebo group (P<0.05). The BBB scores of the E2+LY294002 group were lower than those of the E2 group (P<0.05). Estrogen thus appears to exert a protective effect on spinal motor neurons following OGD, via GPR30. The PI3K/Akt pathway may be one of those involved in the estrogen-related antiapoptotic effects mediated by GPR30.

## Introduction

Spinal cord injury (SCI) may result in severe dysfunction in motor neurons (1,2). The protection of spinal motor neurons following SCI is an important area of research (3–5). However, despite a degree of theoretical progress, there is a lack of effective drugs that are able to improve the motor function of patients following SCI (6–9). The protective effect of estrogen on the central nervous system via the estrogen receptor (ER) has been reported in a number of studies. For example, the use of the ERα ligand, also termed E2, in the treatment of experimental autoimmune encephalomyelitis may reduce the severity of this condition (10). E2 may also reduce ATP-mediated calcium influx into the primary sensory neurons of mice (11). Furthermore, E2 may reduce apoptosis in rat astrocytoma cells via the ER (12). Epidemiological studies have shown that the probability of females developing SCI is lower than that of males, and that the degree of neurological recovery in females is better than in males. Animal experiments have confirmed that estrogen improves motor function in the limbs of injured animals (13,14). However, there are numerous ethical issues in the clinical administration of estrogen, due to its multiple side effects. Thus, further investigation into the neuroprotective effects of estrogen is required, in order to identify novel targets for clinical intervention. The specific mechanisms underlying estrogen neuroprotection following SCI remain unclear. ERs are located on the surface layer of the dorsal horn of the spinal cord, which contains sensory motor neurons, while they are not found in the ventral horn of the spinal cord, which contains motor neurons (15,16). Nonetheless, an improvement in motor function with estrogen treatment following SCI has been observed in animal models as well as in clinical studies (1). It has been shown that estrogen is an antagonist of excitatory AMPA-mediated toxicity in spinal motor neurons via the indirect action of ER-containing glial cells (17).

G-protein-coupled receptor 30 (GPR30) is a membrane-associated estrogen receptor that was originally identified in the 1990s. Its mode of action and effects are different from the conventional nuclear receptors, ERα and ERβ, and it has no homology to these receptors. Thus, GPR30 is a novel estrogen receptor with an independent effect. Previous studies by this group have shown that estrogen improves the motor function of rats with SCI and reduces apoptosis in the spinal cord following SCI, via the membrane receptor, GPR30, rather than the conventional ERs (18). GPR30 receptors are located in the ventral horn of the spinal cord (18), while the classic nuclear ERs are located in the spinal dorsal horn (19). This suggests that the effect of estrogen, mediated by GPR30, on spinal motor neurons may be an important target for neuroprotection following SCI.

In the present study, spinal motor neurons were used to establish cell damage and animal injury models. E2, G1, G15 and LY294002 were used as intervention treatments in order to observe the protective effects of estrogen through GPR30 on spinal motor neurons, and to explore the mechanisms underlying its effects.

## Materials and methods

### Culture of spinal motor neurons

In accordance with previous literature (20) as well as our own experience, spinal motor neurons (Sciencell, Carlsbad, CA, USA) were transported to the laboratory frozen in liquid nitrogen. After thawing at room temperature, the neurons were homogeneously inoculated, at a density of 600–700 cells/mm^2^, in a cell culture apparatus coated with poly-Lysine (Sigma-Aldrich, St. Louis, MO, USA) in medium composed of 482.75 ml Neurobasal medium, 10 ml B-27 (Gibco Life Technologies, Carlsbad, CA, USA), 5 ml fetal bovine serum and 1.25 ml GlutaMAX stock (Gibco Life Technologies; pH 7.0). The medium was changed 8 h after inoculation. A second change was performed at 48 h. At 72 h following inoculation, the cultured neurons were observed.

### Immunofluorescence

Samples were washed with phosphate-buffered saline (PBS) for 10 min and then fixed with 4% paraformaldehyde for 20 min. The fixed samples were then washed twice with PBS and 0.3% triton X-100 was added for 10 min, in order to permeabilize the cell membrane. The samples were then washed twice for 10 min with PBS, blocked with 800 *µ*l blocking buffer for 60 min, and the primary antibodies (mouse anti-rat against SMI32 1:1,000 (monoclonal; Covance, Princeton, NJ, USA) or rabbit anti-rat against GPR30 1:400 (sc-48525-R; polyclonal; Santa Cruz, Dallas, TX, USA) for 2 h at 37°C was added. Following incubation with the primary antibodies, the samples were washed 5 times, for 5 min each time with PBS. The secondary antibodies (rhodamine-conjugated goat anti-rabbit and FITC-conjugated goat anti-mouse) were then added and the samples were incubated at 37°C for 1 h. Finally the samples were washed twice, for 5 min each time with PBS, following which, DAPI was used to stain the nucleus for 10 min. Following DAPI staining the samples were washed 4 times, for 5 min each time with PBS and mounted with anti-quenching resin. The cells were observed by fluorescence microscopy (Olympus MF53; Olympus, Tokyo, Japan).

### Cell treatment

Estrogen E2 (17β-estradiol, Sigma-Aldrich); GPR30 agonist, G1 (Sigma-Aldrich); GPR30 inhibitor, G15 (Tocris Bioscience, Ellisvill, MI, USA); and the phosphatidylinositol 3-kinase/protein kinase B (PI3K/Akt) pathway inhibitor, LY294002 (Cayman), were dissolved in dimethyl sulfoxide (DMSO; Sigma-Aldrich) and added to the medium at the following concentrations: E2 (1, 10 or 100 nM, or 1 *µ*M), G1 (10 nM), G15 (10 nM) and LY294002 (10 nM). Equal amounts of DMSO were added as negative controls.

### Establishment of the oxygen-glucose deprivation model (OGD model)

The cells were place in an incubator containing 95% nitrogen and 5% CO_2_. The original culture medium was replaced by glucose-free Dulbecco’s modified Eagle’s medium (DMEM) solution (Gibco Life Technologies). Following 3 h of OGD, the glucose-free DMEM was changed for the original culture medium and the cells were placed back into an incubator containing 5% CO_2_ and 95% air at 37°C. Following an additional culture period, the corresponding detection indices, including MTT assay, flow cytometry and western blotting, were performed.

### MTT assay procedure

Neuronal growth was detected using a MTT assay. MTT (Sigma-Aldrich, 50 mg) was dissolved in 10 ml PBS. Following sterile filtration, this solution was stored at −20°C for subsequent use. Once grouped, the cells were cultured in 96-well culture plates. Following a cell culture period of 12, 24, 36 or 48 h, 20 *µ*l of MTT was added into each well and the cells were cultured for an additional 4 h. After 4 h, unabsorbed MTT was removed and 150 *µ*l DMSO was added to each well to dissolve any purple crystals. Following 10 min shaking, the samples were placed into a microplate reader (Tecan, Mainz, Germany) in order to measure the optical density at 570 nm.

### Flow cytometry

Cells were collected and centrifuged at 1000 × g for 10 min at 4°C. The supernatants were discarded and 1 ml of ice-cold PBS was added and gently shaken to suspend the cells. The cells were then centrifuged again at 1000 × g for 10 min at 4°C, and the supernatants were discarded. The cells were resuspended in 200 *µ*l of Banding buffer (Roche, Basel, Switzerland). To this buffer, 10 *µ*l Annexin V-FITC and 10 *µ*l PI were added and gently mixed for 15 min at room temperature in darkness. Flow Cytometry was used in order to detect cell apoptosis and to calculate the percentage of cells in early late and total apoptosis, and of necrotic cells in each group.

### Western blot analysis

Following homogenization, the samples were centrifuged at 28341.3 × g for 1 min at 4°C (10). The supernatants were boiled for 5 min. The samples were separated using an SDS-PAGE gel containing 7.5% polyacrylamide. The protein bands were transferred to PVDF membranes (GE Healthcare). The membranes were blocked with 5% non-fat milk in Tris-buffered saline with Tween-20 for 1 h at room temperature and incubated with anti-GPR30 (1:400; Santa) or anti-Akt (1:1,000; KangChen, Shanghai, China) antibodies at 4°C overnight. This was followed by incubation with the appropriate secondary antibodies. Immunoreactivity was detected using enhanced chemiluminescence (ECL; GE Healthcare, Buckinghamshire, United Kingdom) after washing with TBST. Finally, the ECL-exposed films were digitized. Densitometric quantification was performed using ImageJ software (National Institutes of Health, Bethesda, MD, USA).

### Animal models and interventions

Healthy male Sprague-Dawley rats (weight, 200–220 g; The Third Military Medical University, Chongqing, China) were selected and anesthetized by intraperitoneal injection of 5% chloral hydrate (400 mg/kg; The Third Military Medical University). Rats were placed in the prone position on the operating table following back shaving and routine disinfection. Following T8-centric longitudinal cutting of the skin and subcutaneous tissues, the paraspinal muscles were dissected to expose the spinous process and vertebral plate, using ophthalmic scissors to cut the spinous process of the T8, and a hemostat to break the vertebral plate of the T8 along the intervertebral space in order to fully expose the T8 spinal cord. Sterile cotton was used to achieve hemostasis, and a 10 g rod was allowed to fall freely from a height of 1.0 cm in order to induce SCI. The diameter of the lower end of the rod was 2.5 mm. This was able to produce an injury with 10 gcf of energy. Following the injury, the paraspinal muscles and the skin layers were sutured, the wound was disinfected and the rats were placed under a lamp to warm prior to awakening. The rats were then housed and fed in single clean cages at room temperature (20±2°C), with a light/dark cycle of 12 h, and a background noise level of 40±10 db. The cages were frequently cleaned. Nutrition was enforced, and when necessary artificial urination and defecation were employed.

Animals were dosed via the tail vein according to the previous literature (21–24). All drugs were dissolved in DMSO and administered once 15 min and 24 h following SCI as follows: E2 (100 *µ*g/kg), G1 (50 *µ*g/kg), G15 (100 *µ*g/kg), and LY294002 (250 *µ*g/kg).

All treatment procedures were approved by the Institute of Animal Ethics of the Chongqing Southwest Hospital (Chongqing, China).

### Basso, Beattie, Bresnahan (BBB) scoring

Rats were grouped into the following groups: Control, DMSO, E2, G1, E2+G15 and E2+LY294002, with five rats in each group. The average score was calculated from the scores of the individual rats. Animals were placed on a 2 m-diameter flat, smooth area of ground and allowed to move freely. The BBB open space movement score was conducted by two individuals familiar with BBB scoring, who were not participating in the present study (25). The analysis was double-blind. The rats were independently observed and recorded for 4 min in order to determine the number of motions of their hindlimb joints, their movement range and load level, coordination of the forelimbs and hindlimbs, and the activities of front paws, hind paws and tails. Their BBB scores were averaged. There was no filling of the bladder, perineal inflammation, or hindlimb trauma.

### Statistical analysis

Statistical analyses were performed using SPSS 19.0 (SPSS, Inc., Chicago, IL, USA). The data are expressed as the mean ± standard error. Statistical comparisons were performed using unpaired Student’s t-test or one-way analysis of variance. P<0.05 was considered to indicated a statistically significant difference.

## Results

### Estrogen has a protective effect on spinal motor neurons, via GPR30, following OGD injury

**Figure 1:**
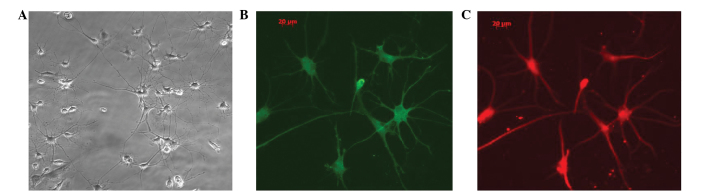
Culture and identification of spinal motor neurons, as well as determination of GPR30 expression in these cells (×200). (A) Spinal motor neurons thawed after 72 h. A number of projections may be observed and visible links exist between the projections. (B) and (C) Double immunofluorescent staining: Green represents the motor neuron marker, SMI32, while red represents GPR30. GPR30, G-protein-coupled receptor 30.

**Figure 2:**
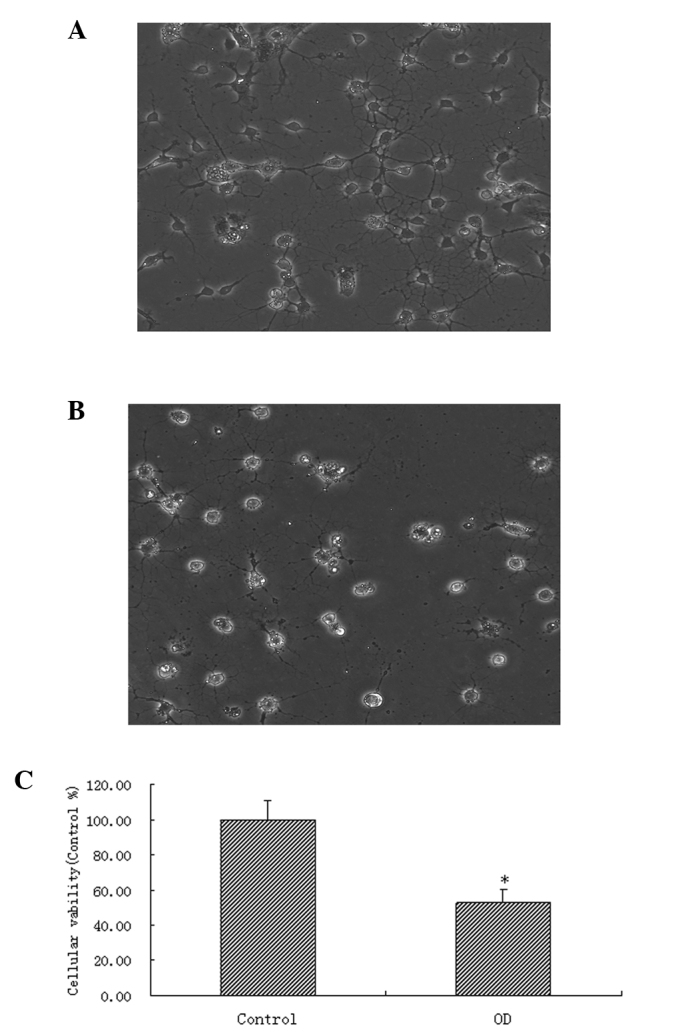
Establishment of a model of OGD. (A) Spinal motor neurons thawed after 72 h, with normal cell bodies and numerous continuous projections (×200). (B) Cells following OGD injury, with various degrees of cell body swelling and breakage of projections (×200). (C) MTT assay of cell viability in each group.^*^P<0.05, compared with the control group. OGD, oxygen and glucose deprivation.

**Figure 3:**
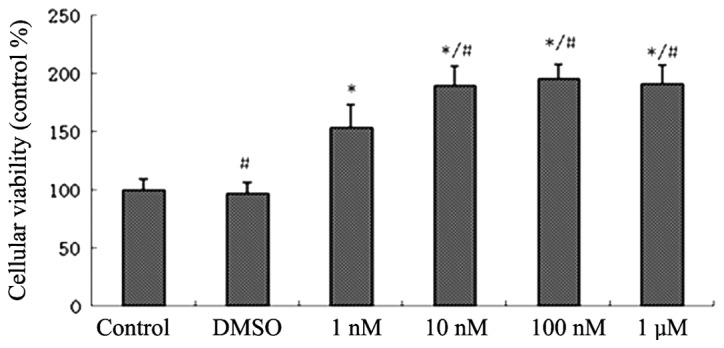
Dose-effect association of the protective effect of estrogen on spinal motor neurons. The MTT assay showed that the cell activity of the 1 nM, 10 nM, 100 nM and 1 *µ*M groups was higher than that of the DMSO group. The cell activity of the 10 nM and 100 nM and 1 *µ*M groups was higher than that of the 1 nM group. ^*^P<0.05, compared with the DMSO group and ^#^P<0.05, compared with the 1 nM group. DMSO, dimethyl sulfoxide.

After the spinal motor neurons were cultured (Fig. 1 A–C), cell injury was induced (Fig. 2 A–C) by OGD. The cell activity after 24 h was detected by MTT assay. In accordance with the previous literature (26), various concentrations of estrogen in DMSO (1, 10 or 100 nM, or 1 *µ*M) were added to the medium as interventions for 24 h in order to observe the protective effect of E2 on spinal motor neurons. The cell viabilities of the E2 groups were higher than those of the DMSO groups. The cell viabilities of the 10 nM, 100 nM and 1 *µ*M groups were higher than the viability of the 1 nM group. No significant differences were detected among the other three groups (Fig. 3). These results suggest that estrogen may exert a protective effect on rat spinal cord motor neurons following OGD.

**Figure 4:**
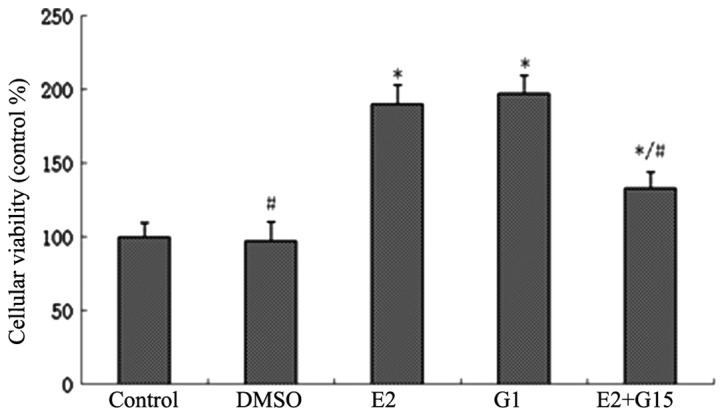
MTT assay results. Cell activity was higher in the E2 and G1 groups, compared with the DMSO group. No significant difference was observed between the G1 group and the E2 group. The cell activity of the E2+G15 group was higher than that of the DMSO group, but lower than that of the E2 group. ^*^P<0.05, compared with the DMSO group and ^#^P<0.05, compared with the E2 group. DMSO, dimethyl sulfoxide.

**Figure 5:**
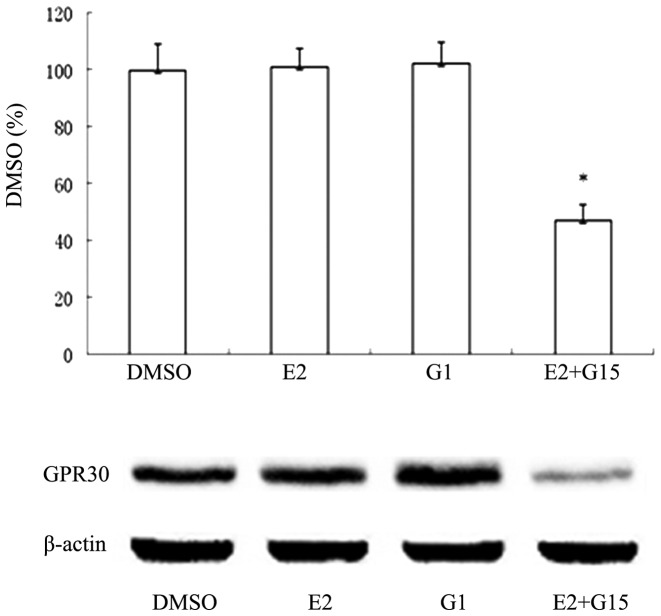
Western blotting results. GPR30 expression in the E2+G15 group was lower than that in the DMSO group, while no significant difference was observed among the DMSO, E2 and G1 groups. ^*^P<0.05, compared with the DMSO group. DMSO, dimethyl sulfoxide; GPR30, G-protein-coupled receptor 30.

In order to understand whether E2 exerts a protective effect via GPR30, the GPR30 agonist, G1, and the GPR30 inhibitor, G15, were used 24 hours after injury. The cells were grouped as follows: Control, DMSO, E2, G1 and E2+G15. The MTT assay showed that the cell viability of the G1 and E2 groups was significantly higher than that of the DMSO group, while no significant difference was detected between the G1 group and the E2 group. The cell activity of the E2+G15 group was higher than that of the DMSO group, while it was significantly lower than that of the E2 group (Fig. 4). This indicated that the GPR30 agonist, G1, exerted the same cell protective effect as E2, while the GPR30 inhibitor, G15, partially inhibited the neuroprotective effect of E2. In order to determine whether G15 indeed inhibited the GPR30 receptor, total protein was collected from the cells following the MTT assay, and samples were analyzed using western blotting. The expression of GPR30 was higher in the DMSO, E2 and G1 groups than in the E2+G15 group (Fig. 5). These results suggested that E2 exerts its neuroprotective effect on spinal motor neurons following OGD, via GPR30.

### E2 protects spinal motor neurons via inhibition of apoptosis

**Figure 6:**
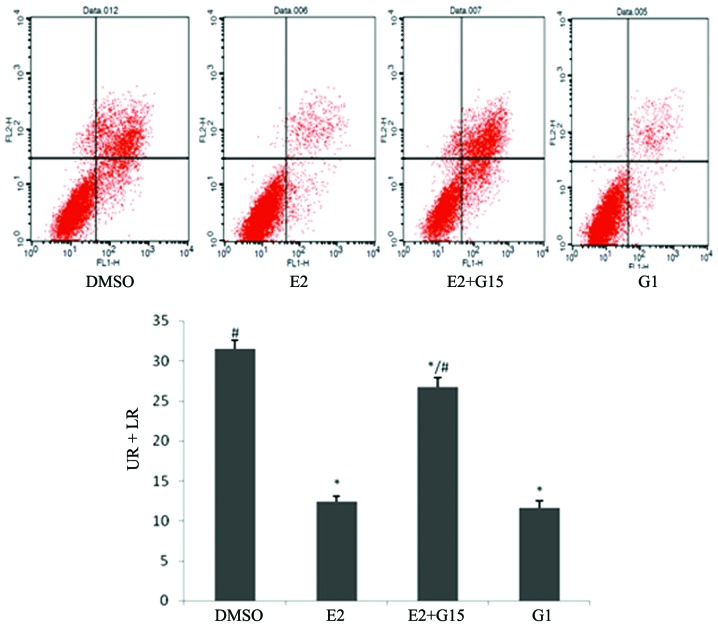
Flow cytometry detection of the proportions of apoptotic spinal motor neurons in each group following OGD (UR + LR). The proportion of apoptotic cells in the E2 group was lower than that in the DMSO group. The proportion of apoptotic cells in the E2+G15 group was higher than that in the E2 group. ^*^P<0.05, compared with the DMSO group and ^#^P<0.05, compared with the E2 group. OGD, oxygen glucose deprivation; DMSO, dimethyl sulfoxide; UR, upper right quadrant with advanced stage apoptosis; LR, lower right quadrant with early stage apoptosis.

Following treatment with DMSO, E2, G1 or E2+G15 in cells subjected to OGD for 24 h, flow cytometry was employed. The results showed that the proportions of apoptotic cells in the E2 and G1 groups were significantly lower than that in the DMSO group. The proportion of apoptotic cells in the E2+G15 group was significantly higher than that in the E2 group (Fig. 6), indicating that E2 exerted an antiapoptotic effect in spinal motor neurons following OGD, via its effect on GPR30.

### PI3K/Akt is the intermediate pathway for the GPR30-mediated antiapoptotic effect of estrogen

**Figure 7:**
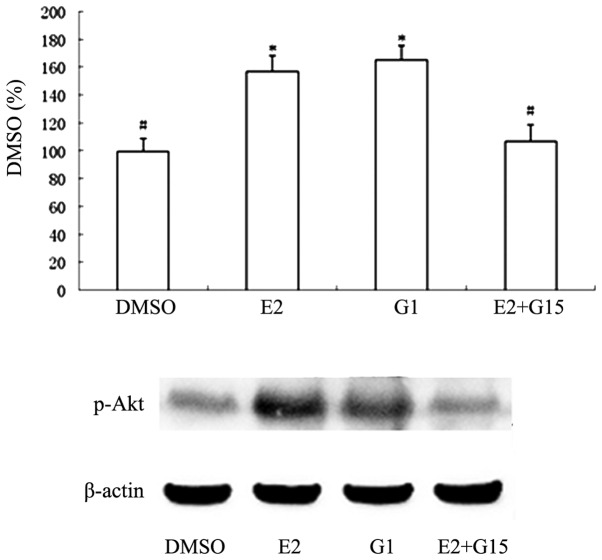
Western blotting results. P-Akt expression in the E2 and G1 groups was higher than that in the DMSO group. P-Akt expression in the E2+G15 group was lower than that in the E2 group. ^*^P<0.05, compared with the DMSO group and ^#^P<0.05, compared with the E2 group. DMSO, dimethyl sulfoxide; P-Akt, phosphorylated protein kinase B.

**Figure 8:**
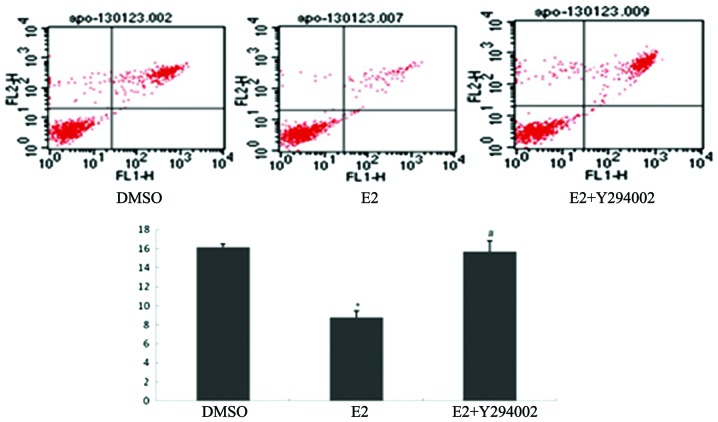
Flow cytometry was used to detect the proportions of apoptotic spinal motor neurons (UR + LR). The proportion of apoptotic spinal motor neurons in the E2 group was lower than that in the DMSO group. The proportion of apoptotic spinal motor neurons in the E2+LY294002 group was higher than that in the E2 group. ^*^P<0.05, compared with the DMSO group and ^#^P<0.05, compared with the E2 group. DMSO, dimethyl sulfoxide; UR, upper right quadrant with advanced stage apoptosis; LR, lower right quadrant with early stage apoptosis.

Western blotting was used to detect the expression of phosphorylated Akt (P-Akt) and its downstream products, using the same treatment groups. The results demonstrated that P-Akt expression in the E2 and G1 groups was higher than that in the DMSO group, while the P-Akt expression in the E2+G15 group was significantly lower than in the OGD+E2 group (Fig. 7), indicating that estrogen regulates the activity of the PI3K/Akt pathway through GPR30. In order to determine whether the PI3K/Akt pathway mediates the connection between E2 and spinal motor neuron apoptosis, the PI3K/Akt pathway inhibitor, LY294002, was used to treat the cells. Flow cytometry showed that the E2+LY294002 group exhibited a higher proportion of apoptotic cells than the E2 group (Fig. 8), indicating that blocking the PI3K/Akt pathway weakens the antiapoptotic effect of E2. This suggests that PI3K/Akt is an intermediate pathway in the GPR30-mediated antiapoptotic effects of estrogen.

### In vivo experiments

**Figure 9:**
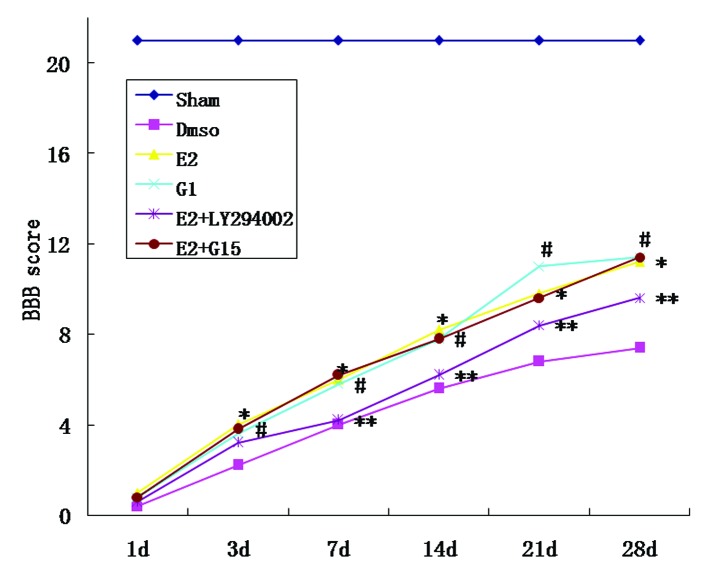
BBB score results. The scores of the E2 group were higher than those for the DMSO group, at 3 days following SCI (^*^P<0.05; E2 group, vs. DMSO group). The scores of the G1 group were higher than those of the DMSO group, at 3 days following SCI (^#^P<0.05; G1 group, vs. DMSO group), with a higher score on day 21 than that of the E2 group (^#^P<0.05; G1 group, vs. DMSO group). The scores of the E2+LY294002 group at 3, 14, 21 and 28 days following SCI were higher than the scores for the E2 group (^**^P<0.05; E2+y294002 group, vs. DMSO group). The scores of the E2+G15 group were similar to those of the E2 group. BBB, Basso, Beattie, Bresnahan score; DMSO, dimethyl sulfoxide; SCI, spinal cord injury.

The appropriate drugs were given to the rats following SCI via tail vein injection with either DMSO, E2, G1, Y294002 or G15. BBB scoring was used to evaluate the motor ability of the hindlimbs of the rats. Estrogen and GI treatments improved the hindlimb motor ability of rats following SCI. The G1 group exhibited a significantly higher BBB score than the E2 group at 21 days. The scores at the majority of time points in the E2+LY294002 group were higher than those in the DMSO group, while the scores at all time points after 3 days were lower than those in the E2 group (Fig. 9), which suggested that LY294002 partially antagonized the neuroprotective effect of E2. The scores of the E2+G15 group exhibited a similar trend to those of the E2 group.

## Discussion

A previous study by this group confirmed that estrogen exerts a neuroprotective effect following SCI, via GPR30 (18). It was hypothesized that apoptosis within the spinal cord may increase following SCI, and that estrogen administration may decrease this apoptosis. In the present study a direct protective effect of estrogen on spinal motor neurons was observed and the mechanisms underlying this effect were subsequently further investigated.

After cells were thawed and cultured to stability for 72 h, they were confirmed as spinal motor neurons by the neurofilament marker, SMI32 (Fig. 1). In addition to mechanical trauma, ischemia and hypoxia are the primary pathological processes that occur following SCI, the persistence of which has been confirmed in observations during clinical autopsy and in microvasculature perfusion following SCI, in animals in which the neuron-containing gray matter of the spinal cord dominates (27). These secondary processes contribute the evolution of the pathological changes of rats with spinal cord injury involving mainly the grey matter (28,29). A linear correlation between the severity of SCI and loss of blood flow in the spinal cord has been demonstrated (30). In the present study, a classical model of OGD was established in order to simulate hypoxic-ischemic injury following SCI. This model was easy to establish, controllable and reproducible. In the current study, marked neuronal swelling was observed following OGD. In addition, the neuronal projections were beaded, with a number exhibiting breakage. These morphological changes were consistent with the changes in cell morphology that are commonly observed following SCI (26). The MTT results also confirmed that cell activity following OGD injury was lower than that in the control group (Fig. 2).

As there have been no previous studies showing that estrogen acts directly on cultured spinal motor neurons, a series of gradient concentrations of estrogen were designed in the present study, with reference to the concentrations used in previous literature for hippocampal neurons (31–33). The concentration of estrogen was varied in order to examine its protective effect on spinal motor neurons following OGD injury. The MTT results demonstrated that only a concentration of estrogen >1 nM exerted a protective effect on cells. Concentrations of estrogen ≥10 nM significantly increased the percentage of viable cells, compared with the 1 nM group, with no significant difference among the three groups treated with the higher doses (Fig. 3). This finding was consistent with the estrogen concentrations that have been shown in previous studies to be effective in neurons in other locations and with other types of injury (31–33). G15 is a recently identified competitive antagonist of GPR30, which interacts specifically with GPR30 and has no affinity for the classic ERs. G15 has become an effective and convenient tool for use in GPR30 studies (34). By using the GPR30 agonist, G1, and the GPR30 antagonist, G15, it was observed that G1 exerted the same cell protective effect as estrogen, while G15 partially offset thIs protective effect (Fig. 4), indicating that estrogen exerts its neuroprotective effect on spinal motor neurons through its action GPR30. These results explain in part why estrogen improves the motor function of animal limbs following SCI (13,14).

In a previous animal study by this group, it was observed that apoptosis in the spinal cord increases in the early stages following SCI (18). It has been reported in a number of previous studies that estrogen may exert an antiapoptotic effect following SCI by enhancing antiapoptotic gene expression, while reducing caspase-3 activity and inhibiting calcium activation (21,35–37). However, the pathways involved in mediating these effects remain to be elucidated. To the best of our knowledge, the present results demonstrate for the first time that estrogen reduces spinal motor neuron apoptosis following OGD by a direct effect on spinal motor neurons, via GPR30 (Fig. 6). This represents a potential approach for exploring the protective effect of estrogen on spinal motor neurons. There are a number of pathways upstream of apoptosis, such as the death receptor-mediated pathway, the mitochondrial pathway and the endoplasmic reticulum pathway (38,39). The PI3K/Akt pathway is involved in the inhibition of apoptosis and the promotion of proliferation in cells, via its effect on the activated state of multiple downstream effectors. It has been observed in a number of studies that GPR30 may regulate the PI3K/Akt pathway. For example, it is involved in estrogen-driven, GPR30-mediated endometrial carcinoma cell proliferation via the PI3K/Akt pathway (40). Furthermore, GPR30 receptors on the endoplasmic reticulum of tumor cells cause rapid calcium mobilization through activation of the PI3K/Akt pathway (41). Finally, using the specific GPR30 agonist, STX, to treat H-38 cells and choriocarcinoma cells significantly activates the PI3K/Akt pathway, thereby increasing intracellular phosphatidyl alcohol, while ERα and ERβ receptor agonists do not produce such effects (42). The present results demonstrated that G1 and estrogen increased the expression of PI3K/Akt pathway metabolites and the phosphorylation of Akt (P-Akt), while G15 partially offset these effects (Fig. 7). In addition, the PI3K/Akt pathway antagonist, LY294002, was used in order to block the protective effect of estrogen on spinal motor neurons (Fig. 8). These results suggest that estrogen exerts an antiapoptotic effect on spinal motor neurons through the regulation of the PI3K/AKt pathway, via GPR30.

In order to investigate whether the above results occur *in vivo*, E2, G1, G15 and LY294002 were administered to rats following SCI, via tail vein injections. E2 and G1 improved hindlimb motor function in rats from an early stage following SCI. LY294002 antagonized the neuroprotective effect of estrogen at a later stage following SCI. No significant difference between E2 gorup and E2+G15 group were observed in the animal studies. This may be related to the following aspects: ER interference cannot be excluded *in vivo*; G15 may pass through the blood-brain barrier; or there may be an impact of the complex internal environment of the body. The antagonism by LY294002 on the protective effect of estrogen, also indicates that the PI3K/Akt pathway is the downstream molecular pathway for GPR30-mediated estrogen neuroprotection.

The present study is only a preliminary exploration of the mechanisms underlying the estrogen protective effect on spinal motor neurons. Due to time limitations, only certain aspects of the mechanisms underlying the effects on apoptosis and the PI3K/Akt pathway were investigated. The interactions of each part of the mechanism require further elucidation.

Estrogen exerts a protective effect on spinal motor neurons following OGD injury. Estrogen exhibits a protective role against apoptosis through its action on the membrane receptor, GPR30. PI3K/Akt is one of the antiapoptotic pathways of estrogen by way of GPR30. Protection of spinal cord motor function following SCI is an important focus in clinical practice, and spinal motor neurons are the primary cells involved in this process. The present study illustrates a preliminary mechanism for the protection effect of estrogen on motor neurons, and identifies targets for clinical intervention that may be further explored in future studies.
